# What Is in Store for EPS Microalgae in the Next Decade?

**DOI:** 10.3390/molecules24234296

**Published:** 2019-11-25

**Authors:** Guillaume Pierre, Cédric Delattre, Pascal Dubessay, Sébastien Jubeau, Carole Vialleix, Jean-Paul Cadoret, Ian Probert, Philippe Michaud

**Affiliations:** 1 Université Clermont Auvergne, CNRS, SIGMA Clermont, Institut Pascal, F-63000 Clermont-Ferrand, France; guillaume.pierre@uca.fr (G.P.); cedric.delattre@uca.fr (C.D.); pascal.dubessay@uca.fr (P.D.); 2 Institut Universitaire de France (IUF), 1 rue Descartes, 75005 Paris, France; 3 Xanthella, Malin House, European Marine Science Park, Dunstaffnage, Argyll, Oban PA37 1SZ, Scotland, UK; sebastien@xanthella.co.uk; 4 GreenSea Biotechnologies, Promenade du sergent Navarro, 34140 Meze, France; developpement@greensea.fr (C.V.); jeanpaulcadoret@greensea.fr (J.-P.C.); 5 Station Biologique de Roscoff, Place Georges Teissier, 29680 Roscoff, France; probert@sb-roscoff.fr

**Keywords:** microalgae, exopolysaccharides, EPS, application, market

## Abstract

Microalgae and their metabolites have been an El Dorado since the turn of the 21st century. Many scientific works and industrial exploitations have thus been set up. These developments have often highlighted the need to intensify the processes for biomass production in photo-autotrophy and exploit all the microalgae value including ExoPolySaccharides (EPS). Indeed, the bottlenecks limiting the development of low value products from microalgae are not only linked to biology but also to biological engineering problems including harvesting, recycling of culture media, photoproduction, and biorefinery. Even respecting the so-called “Biorefinery Concept”, few applications had a chance to emerge and survive on the market. Thus, exploiting EPS from microalgae for industrial applications in some low-value markets such as food is probably not a mature proposition considering the competitiveness of polysaccharides from terrestrial plants, macroalgae, and bacteria. However, it does not imply drawing a line on their uses but rather “thinking them” differently. This review provides insights into microalgae, EPS, and their exploitation. Perspectives on issues affecting the future of EPS microalgae are also addressed with a critical point of view.

## 1. Introduction

**Figure 1:**
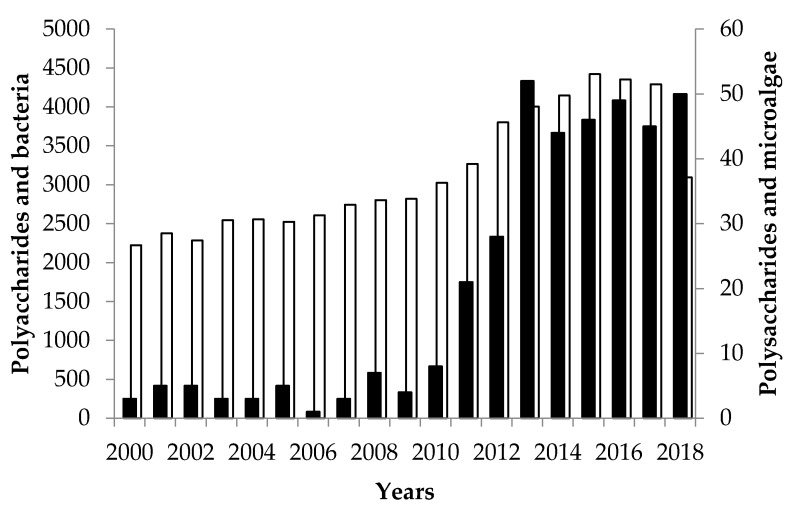
Number of references per year between 2000 and 2018 using the key words “polysaccharides and bacteria” (**☐**) or “polysaccharides and microalgae” (⏹).

Polysaccharides which are the most abundant carbohydrates in nature are high molecular weight biopolymers (10 to 1000 kDa) with complex structures and various physico-chemical properties. They are mainly composed of pentoses and/or hexoses linked by glycosidic bonds leading to linear or ramified homo- and heteropolymers. Depending on the nature of monosaccharides, kinds of glycosidic bonds, presence of some non-sugar constituents (sulfates, organic acid, methyl, amino acids, or amine), they are rigid or flexible macromolecules with various levels of solubility in water. The monosaccharides which are most commonly present in their structures are d-xylose, d-glucose, d-galactose, and d-mannose and some corresponding *N*-acetylaminosugars and uronic acids. Compared to polysaccharides from terrestrial plants, fungi and macroalgae, eukariotic and prokariotic microbial polysaccharides have probably the higher structural diversity [1,2,3]. These microbial polysaccharides have various biological functions. They can be energetic reserve (starch or glycogen), structural components serving structural and protective purposes (β-glucans or peptidoglycan) or excreted outside the cells to form (or not) mucilaginous layers with not well-defined functions. Indeed, some of the roles ascribed to these exocellular microbial polysaccharides also called exopolysaccharides (EPS) are protective barrier, sorption of organic and/or inorganic compounds, binding of enzymes, sink for excess energy, adhesion, biofilm, protection from antimicrobial agents including predators, exportation of cell components, water retention bacteria aggregation, and nutrient source for a bacterial community [3]. The marine and freshwater environments harbor the main part of the microbial biomass on Earth. Among this microbial biomass, microbial photoautotrophs including microalgae and cyanobacteria have been poorly investigated to produce metabolites such as EPS despite their potential. Indeed, the number of microalgae and cyanobacteria species has been estimated to about 800,000 species. Among them only 50,000 have been identified [4,5,6], and less than 100 have been described to produce EPS [2]. These EPS producers are mainly Cyanobacteria rather than eukaryotic microalgae [2,7]. This number is very low compared to of bacteria producing EPS (Figure 1). Note that in the last decade, the number of references on EPS in microalgae are equal to that on bacteria, suggesting that microalgae producing EPS are not so overlooked.

What is the explanation of this lack of knowledge about EPS from microalgae? Probably the best answer is that the culture of these microorganisms is often complex and difficult (notably for eukaryotic microalgae) compared with heterotrophic culture of non-photosynthetic microorganisms. Their generation time is high, most of them are no axenic, some of them are very shear sensitive, the biomass concentration is very low after autotrophic cultures, and some microalgae can require unknown (or poorly controlled) culture conditions (e.g., irradiance and composition of culture media). Moreover, the culture conditions to obtain EPS by microalgae are not always the same as those leading to biomass production. For example, the well-known red marine microalgae, abundantly published for the production of EPS, required specific culture conditions including nitrogen starvation and/or specific N/P ratios to produce exocellular polysaccharides [8]. There are numerous classical culture media that were developed for microalgae maintenance or isolation but not for biomass and metabolites production. The recent emergence of technologies for industrial exploitation of microalgae is currently changing this story. The culture and processing of microalgae is not really a problem of biology but rather a problem of biological engineering. The production of metabolites by microalgae was until recently driven by the third generation biofuels which allowed the development of technologies for large scale microalgae cultures in various culture systems including offshore cultivation, dark systems, open systems (unstirred ponds, race track-type ponds, or circular ponds), and closed systems (vertical tube photobioreactor, horizontal tube photobioreactor, stirred tank photobioreactor, or flat panel photobioreactor) [9,10]. Specific growth modes have been also developed for phototrophic, heterotrophic, and mixotrophic cultivation of microalgae sometimes using wastewaters as source of nutrients [9,11]. After culture, the biomass needs to be harvested for further processing into useable products. In this field, also the development and selection of specific downstream processes including harvesting methods (centrifugation, sedimentation, or membrane technologies), biomass refinery, and purification techniques (chromatography) increase the yield and quality of extracted and purified products. All these technologies decrease significantly the cost of secondary metabolites production by these microorganisms and open the way to the market for some microalgae high value compounds [2,8,12].

Hopefully, outstanding issues still exist. The first one is what is an EPS when we do talk about microalgae? This ostensibly simple question does not find a clear answer in the literature. Indeed, even if the specificities of different exocellular carbohydrates have been well defined for heterotrophic bacteria as capsular polysaccharide, lipopolysaccharides, and EPS [13], they were qualified differently when authors reported data from microalgae (see Section 3.2). The complexity of their structure including sometimes up to 6–7 monosaccharides linked without evident repetition units and substituted by non-sugar groups complicates their characterization using the classical method of glycochemistry [14]. Currently only some EPS from red marine microalgae and Arthrospira species have been the subject of full chemical and rheological characterizations leading to their industrial exploitation in some market niches [8,15]. The increasing of international market demand for natural hydrocolloids and biological agents could be associated in the next future to the development of large-scale cultures of microalgae for original EPS production. This review gives a recent overview on new developments in the field of EPS from microalgae and on their real potential to access to the market in the future.

## 2. Ecology of Microalgae and Diversity

**Figure 2:**
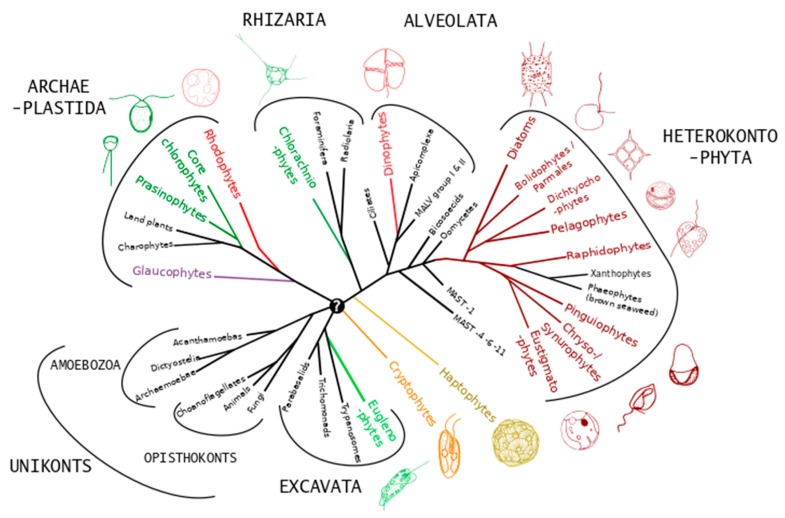
Schematic phylogenetic tree representing the distribution of microalgae (colored lineages with representative images) across major eukaryote supergroups. Adapted with permission from Not et al., Advances in Botanical Research, 64, 1–53; published by Elsevier, 2012 [17].

The term alga (pl. algae) corresponds in fact to a functional group that is most commonly defined as containing all distantly related eukaryotic organisms that have the capacity to perform oxygenic photosynthesis, except “higher” (i.e., vascular) plants. Oxygenic photosynthesis evolved only once during the history of life, in cyanobacteria, leading to the rise of atmospheric oxygen ca. 2.3 billion years ago. Oxygenic photosynthesis first appeared much later in eukaryotes via the primary endosymbiotic incorporation of a cyanobacterium into a unicellular heterotrophic eukaryote host early in the evolution of the Archaeplastida lineage (which now comprises glaucophytes, red and green algae, including higher plants) [16]. At several points during the subsequent history of the Archaeplastida, algae from this lineage have been incorporated via secondary endosymbiosis into heterotrophic hosts from distant evolutionary lineages, forming plastid-containing sub-lineages (i.e., algae) within these groups. In some cases, these secondary endosymbiotic algae have in turn been incorporated as tertiary endosymbiotic plastids in other lineages, or lost their original plastids (in some cases replacing them with alternative endosymbiotic plastids) [16]. This means that algae are taxonomically extremely diverse, having representatives in all but one of the main lineages (“super-groups”) of the eukaryotic tree of life (Figure 2), with most of these lineages containing both algae and non-photosynthetic microorganisms. It also means that all chloroplasts are direct descendants of cyanobacteria, albeit highly modified by massive gene transfer to the hosts, traces of which can be detected in all eukaryotic primary producers. Multicellular algae (= macroalgae or seaweeds) only occur in sub-lineages of the Archaeplastida (green and red macroalgae) and Heterokontophyta (brown macroalgae in the class Phaeophyceae). Microalgae are single-celled algae found in all main eukaryotic lineages except the unikonts (Figure 2).

In line with their extensive taxonomic diversity, microalgae exhibit enormous diversity in terms of their morphology, ecology, and metabolic capabilities.

### 2.1. Morphological Diversity

Microalgae span an exceptionally wide size range (more than three orders of magnitude, ranging from pico-plankton with a diameter of 0.2–2 µm up to meso-plankton that measure 0.2–2 mm), both between and within taxonomic groups. Size spectra can vary in response to succession of life cycle stages or varying environmental conditions. Individual cells of most microalgae are solitary, but many species form chains or colonies. Although exceptions exist, the largest size classes of microalgae are generally dominated by golden-brown groups, notably diatoms and dinoflagellates, whereas the smallest size classes are dominated by green prasinophyte algae. Cell size affects numerous functional characteristics of microalgae. For instance, because of their large surface to volume ratio that facilitates passive nutrient uptake, small cells are particularly well adapted to stable oligotrophic (nutrient poor) waters, whereas larger cells typically perform better in mixed eutrophic (nutrient rich) settings [18].

Seaweeds are unified by the fact that they all have robust carbohydrate-rich cell walls that are of fundamental significance for their survival and often represent the major product of photosynthetically fixed carbon. Microalgal cell coverings, when present, are also most often carbohydrate-based, but are extremely diverse in terms of structure, in several cases also involving some form of biomineralization.

Green microalgae are mostly found within numerous lineages in the phylum Chlorophyta. Prasinophytes are an early diverged group of branching Chlorophyta lineages that include some of the smallest non-motile eukaryote cells (e.g., Ostreococcus, Bathycoccus), as well as larger, mostly motile forms. The cell and flagellar membrane surfaces of larger prasinophytes are often covered with layers of distinctly shaped and often elaborate scales that are produced intracellularly and are primarily comprised of neutral and acidic sugars including 2-keto sugars such as 3-deoxy-lyxo-2-heptulosaric acid (DHA) [19]. Small (‘pico’) prasinophytes are either covered with scales or do not have any discernable matrix (i.e., they are naked). Taxa of the small Chlorodendrophyceae group of green algae consist of motile or non-motile unicells that are covered by a cell wall (or ‘theca’) that is believed to be a product of fused scales comprised mainly of the acid sugars 2-keto-3-deoxy-d-manno-octulosonic acid, 5-*O*-methyl 2-keto-3-deoxy-d-manno-octulosonic acid, and DHA [20]. The Trebouxiophyceae exhibit diverse phenotypes ranging from unicells to colonies to filaments and include also symbiotic green algae of lichens (e.g., Trebouxia). In Chlorella and related genera, the cell wall contains an inner layer of cellulose and in many cases a highly resistant outer stratum consisting of insoluble, acetolysis-resistant, lipid-containing biopolymers termed “algaenans” [21]. In Trebouxia isolated from lichens, the cell wall has low cellulose content and contains β-galactofuranan, a polysaccharide commonly found in fungi [22]. The Chlorophyceae are the largest group of green algae and exhibit significant morphological diversity ranging from motile unicells to large filaments to blade-like thalli. In the Chlamydomonas–Volvox assemblage (i.e., volvocalean flagellates), the cell wall does not contain cellulose but is rather made of a crystalline glycoprotein [23].

The few known unicellular red microalga are exclusively non-flagellated and do not have structured cell walls, but cells are often encapsulated in a sulphated polysaccharide complex. Glaucophytes are a small group of freshwater flagellates, some of which have a cell wall composed of cellulose filaments.

The heterokonts are a very large and diverse group characterized by having two unequal flagella at some point in the life cycle, the longer of which bears tripartite glycoprotein hairs that help to pull the cell along during swimming. Some groups of heterokont microalgae, such as the flagellated raphidophytes or amaeboid chrysophytes, have no cell covering at any stage in their life cycle. Many heterokont microalgae, including eustigmatophytes and pelagophytes, have relatively simple cellulosic cell walls. Silicification occurs in several heterokont lineages. Synurophytes and many chrysophytes are covered with siliceous scales that are produced intracellularly in Silica Deposition Vesicles (SDVs) and extruded to form a composite exo-skeleton [24]. Some dictyochophytes, known as silicoflagellates, produce internal silica skeletons composed of a network of bars and spikes arranged to form a basket-like structure. The Parmales are a group of very small microalgae characterized by production of a cell wall composed of 5 to 8 interlocking silica plates with distinct forms [25]. The Parmales are a sister-group to the diatoms, which are the best-known group of silicifying microalgae. Diatoms have the ability to form a hard but porous silica cell casing, known as a frustule, which can be highly ornamented. Frustule formation occurs in SDVs in proximity to the plasma membrane. The frustule is composed almost purely of silica coated with a polysaccharide layer and usually consists of two overlapping sections. The frustule contains many pores and slits that provide access to the external environment for processes such as waste removal and mucilage secretion.

Dinoflagellates are a diverse group of ecologically important microalgae in the Alveloata phylum, the bi-flagellate cells of which are surrounded by a complex covering called the amphiesma, which consists of continuous outer and inner membranes, between which lie a series of flattened vesicles. In “armored” dinoflagellates, these vesicles contain cellulosic thecal plates, while in “naked” dinoflagellates the vesicles are empty.

The majority of Haptophyta are covered by cellulosic scales that are produced intracellularly in Golgi-derived vesicles. These scales take the form of simple knobs in the Pavlovophyceae and plate-scales, sometimes with spines, in the Prymnesiophyceae. The coccolithophores are a clade within the Prymnesiophyceae in which intracellular calcification occurs on the plate scales, forming the calcareous coccoliths that make up the composite cell covering (the “coccosphere”).

The flagellated cryptophytes have a cell wall called a periplast that consists of a proteinaceous inner layer and surface periplast components with the plasma membrane in between. Both inner and outer layers can be formed of discrete plates that are composed of high molecular weight glycoproteins [26].

The euglenophytes are a photosynthetic sub-lineage of the Excavata (a phylum which contains many unicellular heterotrophic parasitic organisms) that obtained a secondary endosymbiotic chloroplast from the green algal lineage. Euglenophytes have flagella, but also characteristically move by streaming amoeboid movements. Euglenophytes do not have a cell wall as such but have a firm but flexible layer composed of spirally arranged protein strips supported by a substructure of microtubules, termed the “pellicle”. The chlorarachniophytes, another group of microalgae that obtained green plastids by secondary endosymbiosis, typically lack cell walls and have amoeboid cells with multiple pseudopodia.

### 2.2. Ecological Diversity

Microalgae inhabit an extremely wide variety of habitats. They are most commonly found in aquatic environments, both freshwater and marine, but can also be found in a range of terrestrial environments including extreme habitats such as glaciers [27] or deserts [28] and are even found in aerial environments [29]. In addition to cyanobacteria, terrestrial microalgal communities are typically dominated by green algae and to a lesser extent diatoms, which exhibit diverse evolutionary innovations, notably in terms of desiccation tolerance and photophysiology [28]. Encompassing ponds, lakes and rivers, freshwater habitats are diverse, often geographically isolated, and can be extremely variable in terms of physico-chemical parameters such as temperature and pH. Green algae and diatoms tend to dominate freshwater microalgal communities, but certain other microalgal groups such as chrysophytes, dinoflagellates, cryptophytes, and euglenophytes can be well represented. Marine environments are generally more continuous and physico-chemically less variable. In coastal environments, microalgae are abundant in the first few centimeters of benthic sediments. Benthic microalgae belong to various taxonomic groups, but diatoms are generally dominant [30]. In shelf environments, benthic microalgal biomass can greatly exceed that of integrated microalgal biomass in the overlying water column [31]. The diversity of benthic microalgae is typically greater in near-shore intertidal habitats that are subject to high levels of physical disturbance. Microalgae that produce large quantities of extracellular mucous to anchor and protect cells are common in such environments, including haptophytes (e.g., Ruttnera, Chrysotila) and heterokonts (e.g., Sarcinochrysis, Gleochrysis, Glossomastix). In the planktonic realm, eutrophic coastal and continental shelf waters are classically dominated by diatoms, dinoflagellates and calcifying haptophytes (coccolithophores), groups that contain species that have the capacity to form large blooms, while other groups such as non-calcifying haptophytes, cryptophytes, and raphidophytes may produce more localized blooms. Open oceans tend to be dominated by picoplankton groups such as prasinophytes, Chrysochromulina-like haptophytes, and small heterokonts like pelagophytes [32].

Although microalgae are primarily photosynthetic, many exhibit mixotrophic behavior, feeding on prokaryotes or other small phytoplankton in addition to conducting photosynthesis. This has been well characterized for certain dinoflagellates (e.g., Protoperidinium, Dinophysis) and haptophytes (e.g., Chrysochromulina, Prymnesium), but is likely widespread across microalgal diversity [33]. Microalgae, particularly dinoflagellates, can also live in symbiosis with larger heterotrophic protists such as foraminifers or radiolarians, or with invertebrate metazoans such as corals, jellyfish, or acoel flatworms.

### 2.3. Biochemical Diversity

The most conspicuous biochemical differentiation amongst microalgae is in photosynthetic pigment content. In terrestrial environments it is taken for granted that photosynthesis is green. All of the photosynthetic parts of terrestrial plants are green (Chl *a* absorbing in blue and red regions) due to the high quantity of chlorophyll a in their chloroplasts. All algae contain chlorophyll a, but in the aquatic environment, green is not the iconic color of photosynthesis, with golden-brown, yellowish, or even variants of red or blue being common. Chlorophyll is very inefficient for absorbing the green spectrum of visible light, and since light is a scare resource in water, particularly in deeper layers, it is essential for microalgae to harvest the full spectrum of visible light as completely as possible, which they do by having a variety of accessory pigments which absorb light in the wavelengths where chlorophyll is inefficient. While all plastids appear to be derived from a single common ancestor closely related to extant cyanobacteria, a major schism occurred early in the evolution of the Archaeplastida giving rise to two major clades from which all eukaryotic chloroplasts are descended. Members of the clade commonly known as the red lineage contain phycobilins and/or chlorophyll c as the main accessory pigment, as well as abundant carotenoids that have yellow, red, or orange reflectance spectra. Red lineage algae include heterokonts, most dinoflagellates, haptophytes and cryptophytes, each of which have specific carotenoid compositions. The other clade, the green lineage (including green algae, euglenophytes, chlorarachniophytes, and a few dinoflagellates), has chlorophyll b as the main accessory pigment with a much more limited set of carotenoids, hence the typical green color.

In the dark reactions of photosynthesis, carbon dioxide is reduced to carbohydrates via the Calvin cycle. In phototrophs, carbohydrates serve as structural components in cell walls, but also as energy reserves inside the cell. Different groups of microalgae synthesize and store different types of carbohydrates. While cyanobacteria primarily synthesize glycogen (α-1,4 linked glucan), the Archaeplastida gained the ability to synthesize polysaccharides with a far more elaborate structure [34]. Both glaucophytes and green algae synthesize starch, which is an insoluble and semi-crystalline ~1:3 mixture of amylose (unbranched α-1,4-linked d-glucose chains) and amylopectin (α-1,4-d-glucose polymer with frequent α-1,6-branching points), whereas most red algae store an amylopectin-like compound called floridean starch (a α-1,4-linked d-glucose polymer with numerous α-1,6 glucosidic branch points). Red algae and glaucophytes can be distinguished from green algae and land plants by the fact that they synthesize starch in the cytoplasm and not in their plastids. Starch has been retained as the primary reserve substance in some secondary endosymbiotic red lineage microalgae, including dinoflagellates (in which starch is found in the cytosol) and cryptophytes (in the periplastidial space, a compartment derived from the cytosol of the archaeplastidal endosymbiont). By contrast, in several other groups derived from secondary endosymbiotic events β-1,3-glucans have evolved as the major storage polysaccharide, occurring in different forms in haptophytes and heterokonts (chrysolaminarin), euglenophytes (paramylon), and chlorarachniophytes [35]. β-1,3-glucans are generally stored in vacuoles, except in euglenophytes which store highly crystalline paramylon in the cytosol.

Under optimal growth conditions, algae synthesize fatty acids principally for esterification into glycerol-based membrane lipids, which constitute about 5% to 20% of their dry cell weight (DCW). Fatty acids include medium-chain (C10–C14), long-chain (C16–18) and very-long-chain (≥ C20) species and derivatives. Different microalgal lineages have characteristic mono- and poly-unsaturated fatty acid compositions [36]. Under unfavorable growth conditions, many algae alter their lipid biosynthetic pathways towards the formation and accumulation of neutral lipids, mainly in the form of triacylglycerols (TAGs), which can constitute up to 50% DCW [36]. TAGs are typically deposited in densely packed lipid bodies in the cytoplasm of algal cells, although lipid bodies also occur in the inter-thylakoid space of the chloroplast in certain green algae. Hydrocarbons are another type of neutral lipid that can be found in microalgae, generally in low quantities < 5% DCW. Under adverse environmental conditions, the colonial green microalga *Botryococcus braunii* has been shown to be capable of producing large quantities (up to 80% DCW) of very-long-chain (C23–C40) hydrocarbons [2], similar to those found in petroleum. Oleaginous algae can be found among diverse taxonomic groups, and the total lipid content may vary noticeably among individual species or strains within and between taxonomic groups.

## 3. Biomass and Exopolysaccharides from Microalgae

### 3.1. Production of Biomass

**Figure 3:**
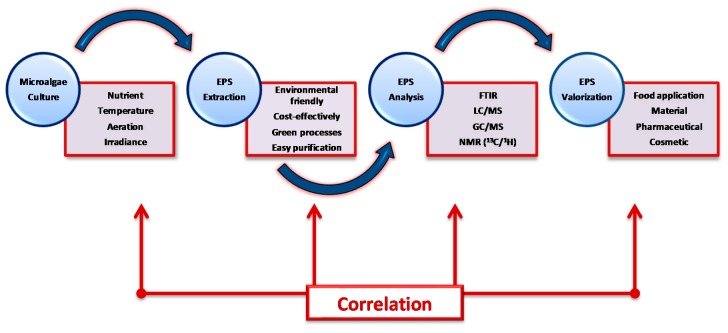
General process strategies to control the production and extraction of exopolysaccharides from microalgae.

As generally described in literature, many parameters must be controlled in order to monitor the growth of microalgal biomass [2,8]. Microalgae can grow in fresh, marine, and highly saline environments [2,37]. Thus, their compositions and capacity to produce metabolites can greatly differ in accordance to several environmental factors, e.g., salinity, pH, mineral content, aeration/CO_2_ supply, temperature, illumination, etc. Optimizing the environmental growing conditions is generally considered as a good way to maximize the biomass production [2,8,38]. For instance, numerous microalgae cannot produce valuable secondary metabolites at high cell-level concentrations and often need depletion in nitrogen and/or carbon sources. Accordingly, specific inductions, which can be distinguished into (i) operating and (ii) nutrient conditions, can increase the synthesis of valuable compounds such as EPS (but not only) under specific stressful conditions such as high salinity, strong light/UV radiation, high temperature, or deprivation of particular nutrients [2,39] (Figure 3). Developing customized processing strategies of microalgal culture is thus of first importance and can lead to efficient biomass production, the ideal being to associate this concept to the greater EPS production (in terms of quality and quantity) which is the real challenging issue [2,3,4,5,6,7,8].

There are typically three categories of microalgae: (i) psychrophilic microalgae growing at temperatures below 17 °C, (ii) mesophilic microalgae which can grow at 20–25 °C, and (iii) thermophilic microalgae growing at temperatures above 40 °C [8,39]. If we focus on EPS-producing species such as the red microalgae belonging to Rhodella or Porphyridium, numerous papers showed a decrease of biomass growth rate under 20 °C [2,8,40]. The quantification of photosynthetic activities of various strains, e.g., *Rhodella violacea, Flintiella sanguinaria,* and *Porphyridium marinum*, highlighted the possibility to estimate their optimum growth temperatures (24 and 28 °C for the last one) [8,41,42]. Regarding other microalgae from extremes environment, it is interesting to mention the optimal biomass growth temperatures around 40 °C for Cyanidium, Galderia or Cyanidioschyzon species (Cyanidiaceae) [43,44,45]. As stated before, the main issue is always to combine both biomass and EPS yields, even if it should be more relevant to talk about EPS productivity (C- g/cells). Lee and Tan [46] showed that increasing the cultivation temperature could stress microalgae (and thus biomass production) in order to produce high value metabolites. This was also observed for red microalgae such as *P. cruentum* and *P. reticulata* with the production of high amounts of EPS, lipids, and/or proteins [2]. Other examples can be easily found in the literature for other EPS-producing microalgae, e.g., for *Spirulina* sp., *Nostoc* sp., *Anabaena* sp. or *Botryococcus braunii* [47,48,49,50].

In addition, the production of microalgae biomass is very dependent to light intensity, i.e., blue (400–500 nm) and red lights (600–700 nm) but also aeration levels. The production of microalgal biomass was thus directly correlated to the photosynthesis activity due to light irradiance under photo-autotrophic conditions [2,8,51]. For instance, the biomass production of *Cyanospira capsulata*, *Porphyridium*, *Synechococcus* sp., or *Arthrospira platensis* was enhanced by high continuous light irradiance [52,53,54,55,56]. However, microalgae can respirate under dark conditions (very low light irradiance) to preserve the metabolic activities which can sometimes lead to the production of specific high-value metabolites such as EPS but often in less quantity [2]. Aeration was described by various authors for increasing the biomass production [47,50,57], but in most of cases, it appeared very hard to correlate the biomass increase with other potential physiological stimuli. Thus, some studies showed that continuous aeration of microalgae culture media can improve (i) the mixture of nutrients (which means enhancing the exchange coefficients in bioreactors), (ii) light diffusion, and (iii) better CO_2_ transfer and O_2_ stripping [51]. Finally, culture media which are traditionally used for microalgae cultivation include Hemerick, Provasoli, BG11 or artificial sea water [2,8,51]. All these media contain the same nutritional elements such as sulphates and phosphorus sources, vitamins (thiamine, biotin, etc.), nitrogen, and metal as traces (iron, magnesium, calcium, etc.). Delattre et al. gave a lot of details regarding the role and impact of some nutrients, i.e., Phosphorus (P), Nitrogen (N), and Sulfur (S) on the production of microalgae biomasses [2]. Note that obviously other valuable metabolites, such as proteins, pigments, and lipids, can be extracted from microalgae but are not the main purpose in this paper. Deep and recent overviews can be found in the literature for microalgae and red microalgae in this context [2,8].

### 3.2. Exopolysaccharides (EPS) from Microalgae

Polysaccharides (PS) from microalgae are, in most cases, considered as primary and secondary metabolites [58]. Their synthesis occurred in Golgi apparatus, excepting for cyanobacteria which is cytoplasmic [59], but we consider that no deep understanding of mechanisms has been published to describe their assemblage by glycosyltransferases and in general with membrane proteins involved at the interface between cytoplasm, inner membrane, and periplasmic space [2]. Note that Rossi and de Philippis [60] published in 2016 a decent paper about chemical features and the role of enzymes and genes but also about hypothetical pathways for apprehending microalgae (exocellular) PS biosynthesis.

For 10 years, microalgae PS have been associated to thousands of papers describing their extraction, purification, structure, and biological (in vitro in most cases) and physicochemical properties. Different intrinsic parameters of structural order seem to be closely involved on the activities reported by authors (the so-called “structure/function” relationship of polysaccharides): (i) flexibility of the macromolecular chain parameter influenced by the nature of the glycoside bonds involved, (ii) nature of the carbon backbone (homopolysaccharide or heteropolysaccharide consisting of neutral oses and/or acids), (iii) molecular weight and mass distribution, and (iv) type of functional groups carried by the macromolecular chain (substitution) groups defined by their position and rate of incorporation.

Today, microalgae PS (and oligosaccharides) are essentially used on the market as moisturizing agents, immune-stimulants, hydrating agents, aggregating agents, plant elicitors (niche markets, selling price ranging from €2.5 k to €200 k/ton) but also for anaerobic fermentation to produce ethanol, hydrogen, organic acids or butanol (>€100 k/ton) [61,62]. With a few exceptions, polysaccharides from microalgae are more considered as by-products of pigments and/or lipids production, often because up and downstream processes are not adapted to their recovering. Then come their high-degree of structural complexity which is another blow to their valorization as high-value molecules. This situation is probably a mistake, and many authors suggest now to focus on strategies for their optimized production, extraction, and purification, which involved to “think” differently the bioprocesses.

PS excreted outside the cells as mucilage or in media are the most studied, probably for technical and economic reasons. The implementation of both extraction and purification procedures is obviously not the same depending on the localization of polysaccharides in the culture, this being particularly true at industrial scale for large volume bioreactors. So far, polysaccharides can be distinguished into three groups, i.e., intracellular (storage polysaccharides), structural, and extracellular. The last ones are in fact more complex and misused in the literature for describing extracellular polysaccharides. Thus, the designation « exopolysaccharides » (EPS) or « extracellular polysaccharides » (ECPS) includes (i) cell-bound (BPS) and (ii) released polysaccharides (RPS) [63]. e.g., EPS for Extracellular Polymeric Substances, which not only include polysaccharides in the literature, and newcomers should keep in mind this setting. As evident for literature, efforts and various methodologies have been made to specifically extract EPS, often dedicated to niche markets (see Section 4). Recent reviews gave a comprehensive and deep analysis of the ways to think and develop the extraction/purification steps [2,8]. Thus, the “chosen” strategy can influence the composition, content, structure, and physico-chemical and biological properties of EPS. From a biorefinery conceptual point of view, authors agree that a unique procedure for extracting and purifying all the metabolites for and at their best is a myth. Factually, choices must be made, and extracting EPS will often be at the expense of other metabolites (see Section 5).

EPS have very complex structures composed up to 8–10 monosaccharides and several substituents as methyl, acetyl or sulfate groups. Here, in contrast to intracellular glucans (starch, chrysolaminarin, etc.) or cell-wall polysaccharides (cellulose-pectin like polymers), this unlimited structural variability is of first interest to identify original biological activities (cosmetic, nutraceutical, and pharmaceutical) and potential as hydrocolloids (food). The complexity is enhanced by other items such as the (i) flexibility of the macromolecular chain; (ii) nature of the carbon skeleton; (iii) mass distribution and molecular weight; (iv) type of functional groups carried by the macromolecular chain (substitution), position, and rate of incorporation; and (v) presence of other covalently-linked molecules, especially proteins.

**Figure 4:**
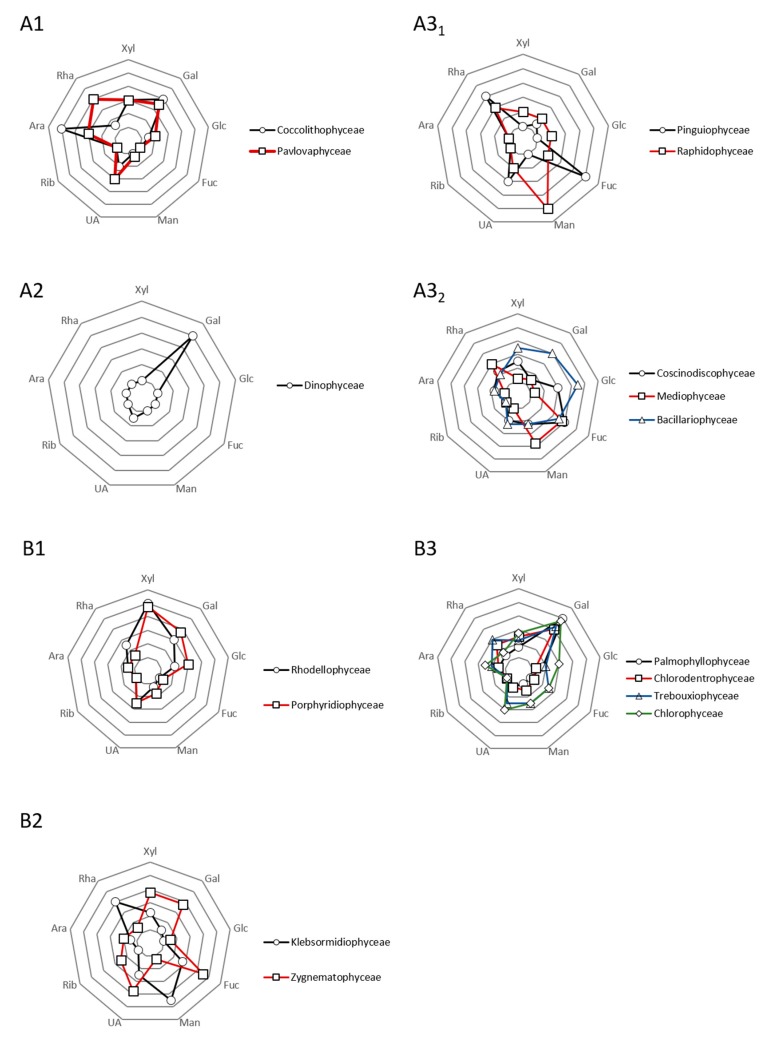
Schematic trends of EPS structural features for various microalgae phyla described in the literature over the last decades. The mappings were compiled according to the data published by [7,8], by attributing 0 to 5 averaged points, regarding the richness of each monosaccharide. **A1**, **A2,** and **A3_1,2_** respectively correspond to Haptophytra, Myozozoa, and Ochrophyta. **B1**, **B2,** and **B3** respectively correspond to Rhodophyta, Charophyta, and Chlorophyta.

Polysaccharides from microalgae can be separated as intracellular, structural, and extracellular glycans. The last category is in fact more complex due to mis- and/or abusive uses in the literature. “Exopolysaccharides” (EPS), “extracellular polysaccharides” (ECP), and polysaccharidic exudates are in, as well as released (RPS) and cell-bound (BPS) polysaccharides. RPS are described as colloidal EPS and low molecular weight exudates (LMW, LMWE, LMWC), and BPS are often sub-divided in sheath, capsule, and slime [2]. Newcomers should keep it mind that the frontiers are very thin between these definitions on paper and the experimental reality in a bioprocess. For three decades, numerous EPS from microalgae have been investigated and that for various phyla, i.e., Charophyta (Klebsormidiophyceae, Zygnematophyceae), Chlorophyta (Chlorodendrophyceae, Chlorophyceae, Trebouxyophyceae), Cyanobacteria, Myozozoa (Dinophyceae), Ochrophyta (Bacillariophyceae, Coscinodiscophyceae, Mediophyceae, Pinguiophyceae, Raphidophyceae), or Rhodophyta (Porphyridiophyceae, Rhodellophyceae). Prybylski et al. [64] recently published an extensive chapter of this EPS heterogeneity, associated with structural data when it was available. More recently, EPS from Haptophyta (Coccolithophyceae, Pavlovaphyceae) were also detailed [65] while these species, such as *Pavlova lutheri* and *salina*, were only described in the past their capacity to produce specific fatty acid [66]. Even if the structural knowledge of EPS is quite “poor”, it seems possible to draw main profile patterns and main monosaccharides and conformation features are often reported in “phylogenetically closed” microalgae (Figure 4).

This statement is less clear for EPS from cyanobacteria which mainly produce Glc-rich EPS with the presence of GlcN/GalN. The heterogeneity of EPS composition between different species belonging to the same genus is also more prominent as reported for many examples (Anabaena, Cyanothece, Leptolyngbya, Microcystis, Nostoc, Phormidium, or Plectonema) [64,65]. Gaignard et al. [65] started the show by screening thousands of microalgae producing EPS, relying on relevant statistical metanalyses (excluding cyanobacteria) addressing the following question “Can we talk about some relationships between phylogeny and microalgae EPS?” Today, a consensus seems to emerge about the influence of extraction and purification procedures, but answering this question could greatly provide significant advances in the understanding of EPS microalgae production, both for industrial uses and also for Evolution comprehension.

## 4. Current Valorization and Markets for EPS Microalgae

In the microalgae world, three main levers enable to put new active ingredients such as EPS on the market [67]. The recent industrial feasibility of EPS production is based on the strain selection, the choice of the adapted photobioreactor, and the strict control of the downstream process. For each EPS and each application [62], food including nutraceutical, cosmetic, and pharmaceutical, these three levels must be studied and adjusted to meet specific regulatory obligations and make profits.

### 4.1. Food and Nutraceutical Applications

Despite a tremendous diversity, the use of microalgae as food in Europe is poor, due to a very constraining European legislation. Europe is under the rule of the Regulation (EC) N° 258/97 that allows the use of microalgae as a food if they appear on the positive list of “traditional” used algae. One must keep in mind that these are European regulations. For other countries i.e., the United States, the positive list differs. As traditional species, i.e., before 15 May 1997, only *Arthrospira platensis*, *Chlorella vulgaris*, *Chlorella pyrenoidosa*, *Chlorella luteoviridis,* and *Aphanizomenon flos-aquae* were authorized. To this list came as novel food a list of few species considered as “novel foods” and as such came through the European administrative tunnel, long in instruction and relatively expensive. These novel food species are *Odontella aurita*, *Ulkenia* sp., *Tetraselmis chui*, *Haematococcus pluvialis*, and Schizochitrium (although, this latter is not seen as an alga [68]). These species authorized in Europe as food ingredients are listed in Table 1.

Some of them are known to produce structural polysaccharides in their cell wall like *Chlorella* sp. and *Tetraselmis* sp. [69,70,71]. However, only the genus Arthrospira is described to excrete a specific EPS called Spirulan [72,73]. *Arthrospira maxima* and *Arthrosprira platensis* are the two most important species cultivated worldwide for nutritional purposes. Associated to these species, phycocyanin appears to be a very valuable pigment used as a natural colorant. The extraction of it leads to the obtention of valorizable co-products and among them polysaccharides. This biorefinery concept is the basis of the European funded Spiralg project (BBI-H2020). SpiralG is a 4-year program aiming at the industrial demonstration of complete valorization of the Arthrospira biomass. In this context, the spirulan appears as a phycocyanin co-product and its industrial production becomes possible and profitable.

### 4.2. Cosmetic Applications

As for the food and nutraceutical field, the cosmetic market is the subject of regulatory constraints, including the Inventory of Existing Cosmetic Ingredients in China (IECIC). This document lists all the ingredients that can be legally used in cosmetics, made or imported in China. Nowadays China represents such a big market that most of the companies developing new cosmetic ingredients work essentially on species mentioned on the IECIC, and this is true for microalgae and cyanobacteria too. *Porphyridium purpureum*, a red microalga, which is well-known to excrete a lot of EPS [74], has the advantage to be China compliant. At the industrial scale, regarding the economic viability of the *Porphyridium purpureum* EPS production, the importance of the strain, the type of photobioreactor, and the technologies used in the downstream process will be described in the two following concrete examples [56,75,76,77].

Silidine^®^ is a mix of oligoelements and small EPS from *Porphyridium purpureum* sold to fight against redness and heavy legs syndrom. Here the use of taylor-made photobioreactor is the key element to, on one hand, innovate and, on the other hand, drastically reduce the culture cost. Indeed, the developed photobioreactor allows applying an oxidative stress to microalgae by closing the air tightly in a culture batch of 1 m^3^.

The second example, Epsiline^®^, is a modified *Porphyridium purpureum* EPS which boost tanning. The selection of an EPS very productive strain and the optimization of the culture parameters in term of media composition, quantity of light, and injection of carbon dioxide enable to reduce by one-third the time it takes to obtain EPS in photobioreactors. The culture stage accounting for a quarter in the cost price of the EPS, this improvement in energy consumption can be allocated to the downstream process. An additional step of hydrolysis permits the creation of a new “medium molecular weight” EPS with a previously unseen biological activity. The selling price of this two proved active cosmetic ingredients is around €100/kg.

### 4.3. Pharmaceutical Applications

To sell pharmaceutical ingredients, manufacturers need a Good Manufacturing Practices (GMP) agreement with very strict articles and with relatively expensive facilities and equipment. In turn, the medical devices market is accessible for medium and small companies and, like the cosmetics field, is rapidly expanding. The literature describes a lot of biological activities which interest this sector [78,79]: antibacterial, antiviral, anti-inflammatory, wound healing, antitumor, antioxidant, etc. [80,81,82,83,84,85,86,87]. EPS from microalgae and cyanobacteria show very interesting rheological properties. Depending to the concentration, they form viscous solutions to gels. Thus, it can be used as active texturizing agents, carriers, or actives protectors and enhancers.

An active ingredient launched as a medical device, like a pharmaceutical product, needs to be very well characterized. The purity and the high microbiological quality are two essential points. To obtain the top grade expected, the use of photobioreactor is compulsory. In addition, disposable photobioreactors permit to perfectly control the EPS production, to guarantee batch reproducibility and microbiological quality. Then comes the tangential ultrafiltration technology as a green process, which allows to drastically purify big molecules like EPS.

Concerning *Porphyridium purpureum* EPS, today medical device grade molecules cost around €150/kg at a concentration of 10 g/L of pure EPS.

## 5. Perspectives on Issues Affecting the Future of EPS Microalgae

As seen previously, the industrial uses of microalgal EPS are still limited to few niche markets with high selling prices like cosmetics. Nowadays, the industrial development of microalgal EPS is mainly limited by the high production costs. In this section, we will focus on the different parameters affecting the development of new products based on microalgal EPS, and we will suggest some potential solutions to overcome them.

In order to better understand why the microalgal EPS production costs are so high, they will be compared to other EPS widely produced at industrial scale by quite similar organisms in quite similar systems, the bacterial exopolysaccharides. Among these exopolysaccharides, we find molecules like dextran, xanthan, or hyaluronan that can be used for many applications like food, cosmetics, health, or bioremediation [88,89,90,91]. Since the production costs are strongly dependent on the microorganism studied, on the culture production system, and on the molecules produced, only overall ideas are illustrated on the following paragraphs.

### 5.1. Differences Between Bacterial and Microalgal EPS Production

Microalgae can be grown in three different ways: in autotrophy, using light and carbon dioxide through photosynthesis; in heterotrophy, using organic carbon source in the dark through respiration like bacteria; and in mixotrophy, using both organic carbon source and light through a mixt process.

#### 5.1.1. EPS Productivity and EPS Concentration

The main difference affecting the EPS production costs between bacteria and microalgae is the EPS productivity. Different reviews concerning microalgae EPS production have been published [2,92]. Both the EPS productivity and the maximum EPS concentration in the culture medium vary significantly depending on the strain studied. Nevertheless, the maximum productivity found in literature for a cyanobacteria (*Anabanea* sp.) is 2.9 g/L/d, for a microalga grown in heterotrophy (*Chlorella* sp.) is 0.36 g/L/d, and for a microalga grown in autotrophy (*Porphyridium* sp.) is 0.19 g/L/d.

The maximum EPS concentrations obtained in the culture medium were 22.3, 5.5, and 1.8 for *Cyanothece* sp. (cyanobacteria), *Botryococcus braunii* (microalgae) grow autotrophically, and *Chlorella* sp. grown heterotrophically, respectively. Nevertheless, average concentrations are most of the time between 0.5 and 1 g/L. Most of these results were obtained in laboratory, with perfectly controlled conditions and are far from the real industrial production conditions where, most of the time, the light cannot be controlled.

In comparison, some *Streptococcus* strains have a hyaluronic productivity higher than 10 g/L/d [62,93], xanthan productivity by *Xanthomonas* is between 2.5 and 10.3 g/L/d [94,95,96,97], and dextran production by *Leuconostoc mesenteroides* can reach 20 g/L/d and even 35 g/L/d [98,99].

Therefore, the bacteria EPS productivity is between 13 and 180 times higher than the Porphyridium EPS productivity, between 7 and 97 times higher than the Chlorella (heterotrophy) EPS productivity, and up to 12 times higher than the Anabanea EPS productivity.

The maximum EPS concentration obtained in the culture medium is 71.5 g/L for *Leuconostoc mesenteroides*, 19.5 g/L for *Xanthomonas arboricola,* and 7 g/L for *Streptococcus equi*. The EPS concentration in the culture medium will affect the downstream processing. This will be discussed later.

#### 5.1.2. Production Systems

When grown in heterotrophy or mixotrophy, the microalgae production is quite similar to the bacteria one. The culture is then performed in a closed fermenter that can be sterilized in order to prevent contamination and where the growing conditions can be controlled [100,101,102,103,104]. For both bacteria and microalgae, the more important parameters that must be controlled are pH, temperature, pressure, O_2_ and CO_2_ (both dissolved and gaseous), and mixing. At industrial scale, very large fermenters (construction cost around $3800 /m^3^) with quite low footprint (around 10 m^2^ for 30 m^3^ production tank) can be used.

When grown autotrophically, the microalgae production differs from bacteria production. Like in heterotrophy the control of the pH, the temperature, the O_2_, and the CO_2_ is required, but light also has to be taken into account. Indeed, to do the photosynthesis, microalgae need to get a good access to light. In most of the industrial microalgae production plants, natural sunlight is used. The control of the light is then tricky. At the beginning of the culture, low-light intensities must be provided in order to prevent photo-inhibition but latter in the culture, high-light intensity is needed to get an optimal growth. Of course, weather conditions will affect the light and so the global productivity of the system but also the other culture parameters like temperature. In some cases, an accurate control of the light is needed in order to maximize the EPS production (stress induction for example). That can be hardly achieved with natural sunlight.

Various production system geometries are used to do so, from open ponds (raceways, circular, etc.) to closed photobioreactors (flat panel, tubular, vertical, horizontal, inclined, etc.) [105,106]. The idea for all these systems is to get as much as possible light to achieve the best productivity. To do so, a compromise between the best ratio S/V (illuminated surface/culture volume) and the capital expenditure of the production system is sought. For example, raceway ponds are quite cheap systems (construction cost around $270/m^3^) but have a low S/V (around 5) while a tubular closed photobioreactor will be more expensive (construction cost around $6750/m^3^) but have a high S/V (around 50) [107,108]. Nevertheless, the quantity of sun light received by a surface is limited. So, the more light you want, the more surface you need. For example, a 30 m^3^ raceway pond (around $8100), considered as a flat horizontal system, will have a total footprint of 120 m^2^. For vertical system, the shadow generated by one system on another must be considered. Thus, 30 m^3^ helical vertical tubular PBR (around $202,000) will have an average total footprint of 375 m^2^. So, for a same working volume, we can see that the footprint of systems using natural sunlight can be 12 to 37 times higher than a fermenter. In places where the access to land is limited, autotrophic production systems are not competitive. In addition to that comes the cost for ground works that increases with the total surface.

Using the EPS productivity data detailed the previous section, we can see that a low footprint 30 m^3^ fermenters could theoretically produce in 3 d, around 900 kg of xanthan (or hyaluronan) or 1800 kg of dextran while a high footprint 30 m^3^ tubular PBR, operated in optimal conditions with Porphyridium during the same period could only produce 17 kg of EPS.

#### 5.1.3. Culture Medium

In heterotrophy, all the nutrients needed by the microorganism are provided by the culture medium. The composition of the later will vary according to the specie produced but it will always contain an organic carbon source (glucose, acetate, etc.), nitrogen, sulfur, micronutrients and vitamins that can be brought under different forms (pure, yeast extracts, etc.) [104]. The cost of the culture medium will be highly related to organic molecules used. It is possible to reduce it by using waste streams or residue [109] (food waste hydrolysate, industrial dairy waste, sugarcane molasses, etc.) instead of buying raw material in bulk.

In autotrophy, the culture medium provides the main macro and micronutrients needed for the growth of the algae. In this case the carbon source is inorganic and can be provided by a direct bubbling of CO_2_ into the production system or by adding carbonates to the culture. The cost of the culture medium is then driven by the nitrogen and the carbon source used. Again, wastewater and residue can be used as well as agricultural fertilizers to reduce the costs.

Some of the microalgae producing the more EPS like Porphyridium or Rhodella are marine species, so they require a seawater-based culture medium to grow. In order to do so, an access to good quality seawater is needed, or artificial seawater medium will be used. Both scenarios add a significant cost to the culture medium preparation.

#### 5.1.4. Downstream Processing

The downstream processing for the recovery of both microalgal and bacterial EPS is quite similar. In most cases, the cells are separated from the culture medium (they can be kept and pasteurized for some microbial EPS) by centrifugation or filtration. In some cases, EPS can then be directly precipitated using ethanol, washed, and then dried. In other cases, EPS can be concentrated and purified by tangential filtration then dried [2,90,95,110,111,112]. For both scenarios, the downstream processing cost will be highly related to the EPS concentration in the culture medium. As seen previously, the EPS concentration in bacteria culture medium can be ten or more times higher than in microalgae culture medium. In this section we will consider microalgae and bacterial culture mediums with EPS concentration of 1 g/L and 10 g/L respectively.

Centrifugation is the most common process used in order to separate the cells from the culture medium containing the soluble EPS. For some bacteria culture, it is necessary to add some water to the culture because the viscosity of the culture is too high (because of the high EPS concentration) to allow the sedimentation of the cells. It is generally not the case for the microalgae culture. Using our example data, we will need to centrifuge 10 times more microalgae culture than bacterial culture to get the same quantity of EPS. The energy consumed to centrifuge 1 m^3^ of culture is estimated at 5 kWh [113,114]. Therefore, 5 MWh will be needed to recover microalgae culture medium containing 1 t of EPS. It will be 0.5 MWh to recover the same quantity but from a microbial culture. Considering an average price of $0.12 /kWh, the centrifugation of the microalgal and of the microbial culture will cost respectively $600 and $60. Recovering microalgae by centrifugation will need a scaling (size, geometry and number) totally different than for bacteria. The initial investment will thus be more important.

If the culture medium is used directly without any concentration steps, 10 times more ethanol at minimum will be used to precipitate microalgal EPS than bacterial EPS. The average cost for one liter of ethanol is around $0.36. So the solvent cost to produce 1 t of bacterial EPS from a 10 g/L culture medium, considering a ratio solvent/culture medium of 1, will be $36,000. This cost will increase up to $360,000 using a 1 g/L microalgal culture medium. This does not consider the cost linked to the solvent regeneration nor all the equipment needed to operate such huge volume of solvent.

Membrane filtration can be used in order to concentrate the EPS before ethanol precipitation or drying. Different membrane geometries (tubular, spiral, etc.) and materials (organic, ceramic, etc.) can be found to do so. To simplify, we will consider here that the filtration performances are the same for both microbial and microalgal culture medium. Because of the difference in EPS concentration, the time or the membrane surfaces needed to concentrate microalgal EPS solution will be much higher than those needed to obtain the same concentration with microbial EPS solution. For example, we are considering here good filtration conditions (permeate flow rate = 80 L/h/m^2^, EPS retention = 100%) for both microbial and microalgal EPS solution and a target EPS concentration of 20 g/L. Concentration factors of 2 and 20 will be needed for respectively the microbial EPS solution and the microalgal EPS solution. If the membrane surface used in both cases is the same, the filtration of the microalgal EPS solution will require twice more time than the filtration of the microbial EPS solution, so twice more energy. In order to get the same filtration time, a doubling of the filtration surface is needed. Doubling the filtration surface will of course have an impact on the membrane investment cost but also on the side equipment like pumps and cooling systems.

In addition, when seawater microalgae species are used, the salt concentration has to be reduced before freeze-drying or precipitating the EPS. This must be done by adding a diafiltration step to the concentration step. The diafiltration step is performed after a first concentration of the EPS solution by adding 3 to 4 diavolumes of distilled water. A final concentration step is then carried out to reach the desire EPS concentration. This diafiltration step consumes large quantities of distilled water and energy. It also generates more wastewater that needs to be treated at the end of the process, increasing again the costs.

#### 5.1.5. Factors Limiting the Development of Microalgal EPS

In the previous paragraphs, the differences between microbial and microalgal EPS production and their impacts on the fabrication costs have been discussed. The microalgal EPS production cost is around 100 times (minimum) higher than the bacterial one. The most important factor is the EPS productivity. Since the microalgae are producing EPS more slowly and at lower concentration than bacteria, bigger cultivation systems are needed to produce the same quantities. If the algae are grown autotrophically using natural sunlight, the capital expenditure related to the production systems is again increased. The main difference concerning the culture medium is related to the use of seawater species that require an access to good quality seawater or to invest in artificial seawater medium. The low EPS concentration in the culture medium will affect the whole downstream processing, from the harvesting to the concentration and the purification of the EPS.

### 5.2. Suggestions to Reduce Microalgae EPS Production Cost

In order to facilitate the industrial development of microalgal EPS, solutions have to be found in order to reduce the production costs. Some suggestions are made in the following sections.

#### 5.2.1. Strain Selections

Thousands of strains are available in the different algae banks all over the world, and even more are still unknown. Most of the strains stored in algae banks have been identified in terms of genus and sometimes species, but only few of them have been characterized in terms of growth rate or specific molecules productivity. As seen previously, the microalgal EPS productivity is the main factor affecting the global EPS production cost. Finding new microalgae producing large quantities of EPS should be a priority to facilitate the industrial development of microalgal EPS. The screening of the strains already available for their potential EPS production would be a good way to identify new promising microalgae that could compete microbial EPS. In order to avoid the desalting need, freshwater strains should be targeted with priority. Such initiatives have already been taken. For example, few hundreds of strains from the Roscoff culture collection have been screened in the French ANR Polysalgue project.

#### 5.2.2. EPS Production and Concentration Improvement

Most of the time, the EPS production mainly occurs during the stationary phase of the microalgae culture. Therefore, when the culture is performed in batch mode, the EPS production will happen after generally few days. In order to reduce this period with no EPS production, changing the production mode to semi-continuous or continuous mode could be a way to maximize the EPS productivity.

A way to increase the EPS concentration and to reduce the downstream costs would be to use an intensified system that can reach high-cell concentration and therefore high EPS concentration, like the ones develop by the laboratory GEPEA in France [115,116]. Of course, the cost of such systems could be higher than that of the more classical ones, so the global techno-economy would have to be checked.

Some strains like Porphyridium [12] or Botryococcus [117] are producing EPS that can partially remain attached to the cells. These EPS are not collected in the culture medium during the harvesting step since they remain with the cells. Processes (bead milling, sonication, etc.) have been developed in order to recover these linked EPS without extracting intra-cellular compounds, increasing so the global EPS production yield [118]. Once again, the cost of these extraction has to be compared to the quantity of EPS extracted in order to validate the economic reliability of this additional step.

#### 5.2.3. Culture Costs Reduction

In order to make the microalgal EPS more economically reliable, a reduction in the culture cost should be made. This is not specifically related to the EPS production.

Nowadays, most of the microalgae production is performed using natural sunlight because it is a free energy source. As seen previously, production systems using natural sunlight could be expensive and have a large footprint. One way to reduce the cost could be to grow microalgae in heterotrophy like bacteria or to use high productivity photobioreactors with artificial light in a context of circular economy. In such system the quantity of light can be monitored according to the microalgae needs, in order to achieve the best productivity with a low footprint. In a circular economy approach, free energy from renewable sources (wind, photovoltaic, etc.) could be used in order to power the system. Xanthella Ltd. is currently developing such system in Scotland within the framework of the ENBIO project (https://aslee.scot/). This would allow the production of microalgae without a lot of sunlight but with a strong potential for renewable energy. The use of by products like wastewater and flue gas could also be investigated as nutrient sources. The impact on the final product quality will have to be checked.

#### 5.2.4. Biorefinery Approach

Another approach, instead of trying to reduce production costs, could be to have a better valorization of all the biomass produced, not only the EPS but also the microalgae cells and the molecules they contain. This biorefinery approach would allow to spread the culture production costs between different products and not only the EPS. This approach has already been studied at lab scale on different strains [12,119,120,121], but the authors are not aware of any industrial examples.

#### 5.2.5. Omic Approach

The identification of EPS producer microalgae and its EPS characterization require a long investment, involving notably time-consuming screening and analysis steps, which are not compatible with industrial requirements. A phylogenic mapping of microalgae correlating both EPS production ability and structure with specific class, genus, or species would be helpful to predict the potential interest of an isolate microalgal strain and their industrial valorization. Although these data are not available, omics-studies may be a relevant approach to establish such data. Notably, genomics and transcriptomics could provide crucial understanding towards EPS production as it was shown for lipids biosynthesis pathways. For example, the exploitation of microalgae genomes database would contribute to the listing and distribution of gene families involved in EPS synthesis (i.e., Glycosyl-transferases), among various selected microalgae models. However, the availability of full microalgae genome sequences is restricted to few microalgae models and hence will not give a significant and relevant mapping. Transcriptomics can offer more exploitable data than the genomics approach because it may inform about EPS synthesis related genes activated in EPS production physiological conditions. The characterization of these genes may really contribute to establish defined expression patterns specific to microalgae phyla, genus, or species. From now on, more than 700 microalgae transcriptomes are available on algaedatabase (www.algaedatabase.org/). Analyses of some transcriptomes have shown the expression of glycosyl-transferase potentially involved in membrane or excreted polysaccharides. A large-scale comparative analysis of transcriptomes, associated to specific phylogenetically representative microalgae, constitutes the more appropriate way to provide a global mapping of EPS producer microalgae, considering particularly technologic skills and tools and the bioinformatics analysis systems for high-throughput data processing.

## 6. Conclusions

Microalgae and cyanobacteria have undergone a long evolution since their apparition on Earth several billions of years ago. They have colonized almost all ecosystems including those without water and are adapted for growing in all types of aqueous environments (fresh, salty, and brackish), temperatures (ranging from tropical to polar environments), pH (0 to 11) and irradiances (surface and deep waters but also symbiosis inside some animals). Being mainly photoautotrophs organisms, a significant part of them are capable of heterotrophic and mixotrophic metabolisms. They are at the origin of the life as known today and provide the cornerstone and the basis of the food pyramid (phytoplankton). This huge taxonomic and metabolic diversity is a reason to be optimist for finding new secondary metabolites in these biomasses. However, even if the recent emergence of technology for the mass production of microalgae has open the way to their industrial exploitation, the costs associated to the production of low values molecules remains prohibitive. The EPS from microalgae make no exception. Although some very original structures linked to interesting rheological properties and/or biological activities begin to be described in literature and patented, their exploitation by industry is in its infancy. This can be easily explained by the analysis of the polysaccharide market where EPS from microalgae must compete with those from terrestrial plant, fungi, macroalgae, bacteria, and animals. In this competition, the sole polysaccharides from terrestrial plants such as cornstarch and cellulose derivatives and those from macroalgae such as alginate or carrageenan account for a big share of the market in chemical, food, and pharmaceutical/cosmetics fields. The microbial polysaccharides exploited from heterotrophic bacteria and fungi such as xanthan or pullulan having higher producing costs have been able to operate in the market because of their intrinsic properties giving them access to space not yet occupied by “low costs plant and macroalgae polysaccharides”. However, the cost ratios between microalgae EPS and those of bacterial or fungal polysaccharides remain higher than a factor 10–100, closing for them the market of texturing agents. Some pharmaceutical and cosmetic high value applications are compatible with the cost of production of microalgae EPS. In the pharmaceutical field, the complexity of marketing authorizations and the need to use well defined polysaccharidic structures with non-variability made this area of exploitation a market not yet available for microalgae polysaccharides. The sole example of animal heparin enthroned as standard anticoagulant despite the numerous published and patented sulfated polysaccharides is proof of the difficulty to ensure this market uptake. The cosmetic field can be considered as mature to welcome polysaccharides of microalgae such as EPS and it is surprising that except some EPS from red microalgae or spirulan from Arthrospira strains, a small part of them has increased value in this field. The future for microalgae EPS could be some other niche markets compatible with high costs of production such as the nutraceutical (nutritional supplements) where some of these EPS could have added value as prebiotic, antioxidant, and other. Using algal EPS for the “green” synthesis of nanomaterials and nanoparticles as well as for removing heavy metals in wastewater treatment should be also included as insights for potential future applications.

## Figures and Tables

**Table 1:** Microalgae authorized for food use in the European Union.

Algae	Year of Approval
*Arthrospira platensis* (spirulina)	Used prior to May 1997
*Chlorella luteoviridis*	Used prior to May 1997
*Chlorella pyrenoidosa*	Used prior to May 1997
*Chlorella vulgaris*	Used prior to May 1997
*Aphanizomenon flos-aquae*	Used prior to May 1997
*Odontella aurita*	2005
*Ulkenia* sp.	2009
*Tetraselmis chuii*	2014
*Haematococcus pluvialis*	2014
*Schizochytrium* sp.	2015
